# Radar Emitter Recognition Based on the Energy Cumulant of Short Time Fourier Transform and Reinforced Deep Belief Network

**DOI:** 10.3390/s18093103

**Published:** 2018-09-14

**Authors:** Xuebao Wang, Gaoming Huang, Zhiwen Zhou, Wei Tian, Jialun Yao, Jun Gao

**Affiliations:** 1 College of Electronic and Engineering, Naval University of Engineering, Wuhan 430033, China; wangxuebao_china@163.com (X.W.); tianwei09@tsinghua.org.cn (W.T.); yjlpaper@outlook.com (J.Y.); gaojunnj@163.com (J.G.); 2 Armed Police Command College, Tianjin 300250, China; mini_paper@sina.com

**Keywords:** radar emitter recognition, energy cumulant, short time Fourier transform, base noise reduction, reinforced deep belief network

## Abstract

To cope with the complex electromagnetic environment and varied signal styles, a novel method based on the energy cumulant of short time Fourier transform and reinforced deep belief network is proposed to gain a higher correct recognition rate for radar emitter intra-pulse signals at a low signal-to-noise ratio. The energy cumulant of short time Fourier transform is attained by calculating the accumulations of each frequency sample value with the different time samples. Before this procedure, the time frequency distribution via short time Fourier transform is processed by base noise reduction. The reinforced deep belief network is proposed to employ the input feature vectors for training to achieve the radar emitter recognition and classification. Simulation results manifest that the proposed method is feasible and robust in radar emitter recognition even at a low SNR.

## 1. Introduction

Radar emitter recognition is an important part in radar reconnaissance and confrontation systems, which is on top of the radar signal sorting [1] and leads the recognition [2,3], location [4], and tracking [5] tasks. The recognition results directly influence the performance of a radar reconnaissance system, so, a lot of recognition studies have been done centered on the five common parameters of radar: radio frequency (RF), time of arrival (TOA), pulse amplitude (PA), pulse width (PW) and angle of arrival (AOA) [6,7,8]. While the electromagnetic environment is more and more adverse, and the signal system becomes more and more complicated, it is not enough to complete the radar emitter recognition only depending on common parameter matching methods. Pulse compression radar provides some performance advantages to face the complicated electromagnetic environment and variable signal patterns with its better anti-interference [9]. Hence, radar emitter recognition based on intro-pulse information has become a new research field. Later, new radar emitter recognition methods that get higher recognition rates and offer more recognition information by analyzing the intra-pulse signal of pulse compression radars were presented [10,11,12]. Our work circles on the research of radar emitter intra-pulse signal analysis in a low-SNR situation to attain a better recognition performance.

A lot of studies on radar emitter recognition based on the intra-pulse features have been conducted. According to the supplied features, these studies mainly concentrate on the time domain features [10,12,13], transformed domain features [10,11,12,13,14] and statistics features [15]. From the aspect of the recognition method, the studies can be divided into two groups: classifiers based on multi-thresholds and multi-regulations [10,12], and classifiers based on learning algorithms [16,17,18,19,20,21]. However, it is one-sided to divide the radar emitter recognition only discussing the recognition features or recognition methods separately, because the recognition features are related to the recognition methods, and the length, dimension, structure and format of the features have decided the optimal recognition methods, so it is a good idea for radar emitter recognition method categories to be defined by combining the features and methods together as follows.

The first category is based on the single continuous feature vector or matrix stemming from a certain transformation and its ramifications, including the time domain autocorrelation, frequency domain transform, time frequency transform, etc. The vectors and matrixes are commonly in large size and cannot directly gives the exact characteristic values, so neural networks, machine learning algorithms and deep learning models are used to extracted the features automatically and then complete the recognition task. In [3,21,22,23], the short time Fourier transform (STFT), Wigner-Ville distribution (WVD) and Choi-Williams distribution (CWD) are used as feature input to convolutional neural network (CNN) for recognition. All three of these achieve good results, while in [3], the network is not modified for the best; in [22], the method refers to the different carrier frequency of the eight waveforms in which the polyphase coding recognition might have little generalization; and in [23], it shows relatively high recognition results without enumerating the concrete researched radar waveforms, which is a vital factor for evaluating any method. The second category is based on discrete multi-characteristic values. These recognition methods gain statistical features and thresholds by calculating the features as the first category shows based on formulas, and they usually complete the recognition task via a multilayer decision criterion. In [10,12], they mainly report achievements in the study of polyphase codes signal classification. Many thresholds for multilayer judgments are needed to calculate this. The third category is based on joint multi-feature vectors and matrices which can increase extra space information of different represented objects. Each component in the joint multi-feature vectors and matrixes is continuous as the first category described. Further, the space information can support the extra features formed by the distributions of vectors/matrixes. Because the foundation of the third category originates from the first one, their recognition and classification methods are similar. In [13,15,21,24], different features are combined together to increase the uniqueness of representation, which makes the recognition easier compared with before. All the recognition methods aim to attain the high-correct recognition rate and good generalization ability, which depends on whether the chosen features have obvious distinction and anti-noise property, and whether the recognition methods have strong adaptability to the data outside the learning set.

In this paper, we are trying to study a novel radar emitter recognition method combining the transformed time-frequency domain feature with a modified deep learning model, reinforced deep belief network (RDBN). It aims to achieve a high recognition rate at a low SNR and have generalization ability for eight types of radar intra-pulse signals at the same intermediate frequency (IF). The paper is organized as follows: the description and hypothesis of the problem is stated in Section 2. In Section 3, the radar emitter intra-pulse signals we studied are displayed. In Section 4, the novel recognition method is explained in detail, including the feature transforming, preprocessing denoising for feature extracting and recognition based on RDBN. And simulations and discussions of the novel recognition method are displayed in Section 5. Finally, conclusions are reached in Section 6.

## 2. Problem Description and Hypothesis

The radar emitter recognition based on intra-pulse features is an important part in the radar emitter recognition research field. When the radar receives the signal, the five common parameters are extracted. The radar recognizes the target by matching these parameters with a prior knowledge base. On this basis, studies on radar emitter intra-pulse feature extraction are organized to provide the precise recognition with more reliable information. Also, obtaining the intra-pulse information is essential in the electronic interference and specific emitter identification (SEI).

As stated in the Introduction section, the radar emitter intra-pulse analysis mainly concentrates on time domain analysis, frequency domain analysis and time-frequency domain analysis, or their variations and combinations. In this work, the STFT is used to get the intra-pulse’s two dimensional time-frequency distribution (TFD), and then an energy cumulant is applied on the TFD result as a new feature. In fact, we have studied STFT and combined it with convolutional neural network (CNN) in [3]., and this also gave good recognition results. However, it did not thoroughly solve the recognition problem in the front-end CNN model until we added a decision criterion to complete the task. The noise at low SNR resulted in deterioration of the STFT images, which made the CNN model extraction of the features of STFT images inefficient. We tried to search for a transform based on STFT to get a feature that has an advantage in recognition work, so in this work, a new feature based on the STFT is discussed. The energy cumulant of short time Fourier transform (EC-STFT) is defined as the input feature vector. With the development of neural networks, machine learning and deep learning [25,26], AI algorithms have gradually become widely used in different recognition fields, such as text, image, speech and signal recognition [27,28,29,30]. Especially, deep learning models are studied a lot for their good performance. In this work, we combine the transformed TFD with modified DBN to achieve a higher recognition of radar emitters. This application can achieve a better recognition result even at a low SNR. 

**Figure 1:**
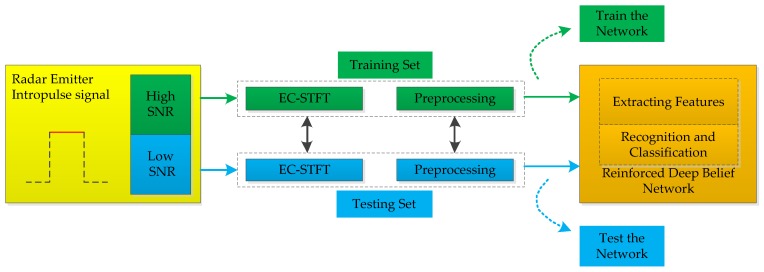
Framework of the radar emitter recognition in this work.

Figure 1 shows the whole framework of the radar recognition method based on EC-STFT and RDBN proposed in this work. The radar emitter intra-pulse signals generated by simulations are divided into two parts, one for training the RDBN, the other for testing. The signals in training set are loaded addictive white Gaussian noise (AGWN) with large SNR, while signals for testing are loaded AWGN with small SNR. Based on this, the RDNB’s adaptive capacity to environment can be examined, and the proposed method’s robustness in this work can be validated. Then the EC-STFT values of intra-pulse signals are calculated. During this step, preprocessing is conducted to ensure the saliency of the new feature and decrease the influence of the noise. Finally, the processed data are used to train the RDBN, and the network will extract their features and complete the recognition task. The test set validates the network’s feasibility and reliability.

To ensure the completeness of the research problem and the applicability of the proposed method, some hypotheses are made as follows:

- The intra-pulse signals stem from sorted signals, and the influence of false alarms and the missed detections are not considered.
- The five common parameters of radar emitter are known a priori. The intra-pulse signal is simplex, and the parameters of the signal remain invariable during the PW.
- This work aims to solve the problem based on the sample database by offline training, so the recognition of radar emitter without database information and the online learning are not considered.

## 3. Radar Emitter Intra-Pulse Signals

The radar emitter intro-pulse signals are truncated in a pulse during the PW from the received pulse train of radars, which can have different modulations. The radar emitter intra-pulse signals considered in our work include: frequency shift keying (BFSK/QFSK), linear frequency modulation (LFM), nonlinear frequency modulation (NLFM), normal signal (NS), phase shift keying (BPSK/QPSK), polyphase codes modulation (FRANK). In our work, we categorized these eight types of signals into two parts according to their similar frequency and phase characteristics.

### 3.1. Frequency Modulated Signal

The BFSK, QFSK, LFM and NLFM are divided into one part for their referring to change of frequency. Simultaneously, BFSK and QFSK are classified as discrete frequency modulation (DFM); LFM and NLFM are classified as continuous frequency modulation (CFM).

#### 3.1.1. Discrete Frequency Modulated Signal

The DFM is more intuitionistic than FSK, for their frequency spectrums are discrete peaks. And their TFDs present finite short discontinuous stripes. At high SNR, these phenomena can be regarded as the DFM’s unequal characteristics. The DFM signals can be written as:

(1) $$x(k)=Aexp(j(2\pi (f_{0}+c_{l}\Delta f_{r})k+\varphi )),$$

where A is the constant amplitude, $f_{0}$ is the initial frequency, $\Delta f_{r}$ is the step frequency and valued according the requirement, $c_{l}$ is the frequency control code, l is the length of $c_{l}$, and φ is the initial phase. And x(k) works as FSK signal when $c_{l}$ takes different values. Suppose Ψ{0,1} is composed by 0 and 1, Ψ{0,1,2,3} is composed by 0, 1, 2 and 3. When $c_{l}\in \Psi \{0,1\}$, the x(k) is defined as BFSK; when $c_{l}\in \Psi \{0,1,2,3\}$, the x(k) is defined as QPSK. Equation (2) gives the definition:

(2) $$\mathrm{BFSK}:\;c_{l}=\Psi \{0,1\}=\{\underset{l}{\underset{︸}{\ldots ,0,\ldots ,1,\ldots }}\},\;\mathrm{QFSK}:\;c_{l}=\Psi \{0,1,2,3\}=\{\underset{l}{\underset{︸}{\ldots ,0,\ldots ,1,\ldots ,2,\ldots ,3,\ldots }}\}.$$

#### 3.1.2. Continuous Frequency Modulated Signal

Both LFM and NLFM’s frequency are continuously changing, and their frequency spectra present modulation bandwidths. While the LFM’s frequency changing rate is constant, the NLFM’s frequency changing rate is gradually increasing, decreasing or disorderly, so the LFM’s TFD shows a long continuous band with unchanging slope, and the NLFM’s is changing. According to the description, the CFM signals can be given by:

(3) $$x(k)=Aexp(j(2\pi (f_{0}+F(\alpha_{i},k))k+\varphi )),$$

where $F(\alpha_{i},k)$ is the function of the modulation slope vector $\alpha_{i}$ and time k. And $\alpha_{i}$ can be composed by different slope values $\alpha_{i}$ (MHz/μs). In this work, $F(\alpha_{i},k)$ is assigned by the polynomial of k to form different CFM signals as follows:

(4) $$\mathrm{LFM}:\;F(\alpha_{i},k)=\frac{1}{2}\alpha_{1}k^{2},\;\mathrm{NLFM}:\;F(\alpha_{i},k)=\frac{1}{2}\alpha_{2}k^{2}+\frac{1}{3}\alpha_{3}k^{3}.$$

### 3.2. Phase Modulated Signal

The phase modulated signals are frequency constant signals with phase jumps which are controlled by the phase codes. In this work, NS, BPSK, QPSK, FRANK are classified as phase modulated signal, and it is given by:

(5) $$x(k)=Aexp(j(2\pi f_{0}k+\Phi_{c_{l}}+\varphi )).$$

The x(k) refers to different phase modulated signals as $\Phi_{c_{l}}$ gets different phases which is given by:

(6) $$\mathrm{NS}:\;\Phi_{c_{l}}=C,\;\mathrm{BPSK}:\;\Phi_{c_{l}}=\Psi \{0,\pi \},\;\mathrm{QPSK}:\;\Phi_{c_{l}}=\Psi \{0,\frac{\pi }{2},\pi ,\frac{3\pi }{2}\},\;\mathrm{FRANK}:\;\Phi_{c_{l}}=\frac{2\pi }{M}(m_{1}-1)(m_{2}-1),m_{1}=1,\ldots ,M;m_{2}=1,\ldots ,M,$$

where C is a constant phase, Ψ{0,π} is composed by 0, π and decided by the phase control code $c_{l}$ (0, 1), $\Psi \{0,\;\frac{\pi }{2},\;\pi ,\;\frac{3\pi }{2}\}$ is composed by 0, $\frac{\pi }{2}$, π, $\frac{3\pi }{2}$ and decided by the phase control code $c_{l}$ (0, 1, 2, 3), and M is FRANK code’s order.

## 4. The Proposed Recognition Method Analysis

### 4.1. Feature Selection and Calculation

#### 4.1.1. Short Time Fourier Transform

**Figure 2:**
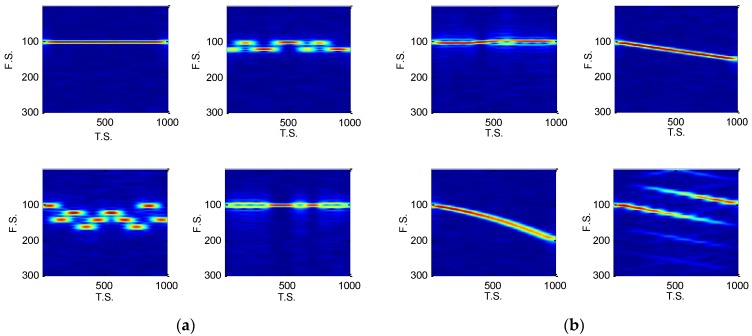
The image displays the TFD of eight type signals at 20 dB: (**a**) STFT of NS, BFSK, QFSK and BPSK; (**b**) STFT of QPSK, LFM, NLFM and FRANK.

The STFT is a widely used time-frequency analysis tool [31]. It converts the time-domain signals into two dimensional TFD, which presents a frequency variation trend with time. Recently, many studies about STFT were conducted, getting good results [32,33,34,35]. In [32], Aubel developed a theory of super-resolution from STFT measurements and presented a new recovery method for pure Fourier measurements. In [33], Mateo proposed a new method to compute the STFT by fixing the window size in the frequency domain instead of the time domain, which is simpler than the exiting method, and the implementation of MatLab functions can be found in [35]. In [34], Torres presented a novel spectral observer with an adjustable constant gain for reconstructing a given signal by means of recursive identification of the coefficients of a Fourier series that performed better than STFT according to the applications. These studies reported good resolution improvement results according to the discussions. Instead of considering the microscopic features of resolution, we think more about the presentation of macroscopic frequency variation trends with time. For that, the resolution improvement was achieved under ideal condition, while our study was conducted at a low SNR which is little influenced by the resolution. In this work, we adopted Auger’s method to compute the STFT [36]. According to [31], the discrete STFT is defined by:

(7) $$STFT_{x}(m,n)=\sum_{k=0}^{N-1}x(k)h^{*}(k-m)exp(-j\frac{2\pi n}{N}k)$$

where h(k) is the time window function, $h^{*}(k)$ is the conjugation of h(k) and the window function’s time shift and frequency shift provide the STFT with localization characteristics, *N* is the length of the x(k). The STFT uses h(k) to intercept x(k) and makes Fourier transform (FT) on the intercepted part. By shifting k, the h(k)’s central position is shifted and the FT at different time is attained, so the STFT compensates the FT’s lack of location analysis capability. In our work, a Hamming Window with a length of N/4 was chosen, the IF of signals was set 10 MHz, the sample frequency was 100 MHz and the PW was 10 μs, so the length *N* is 1000, and the time samples (T.S.) length is 1000, frequency samples (F.S.) length for 500 in the TFD. We cut off some useless part of the TFD images. Figure 2 shows the images of eight types of signals’ STFT at 20 dB.

**Figure 3:**
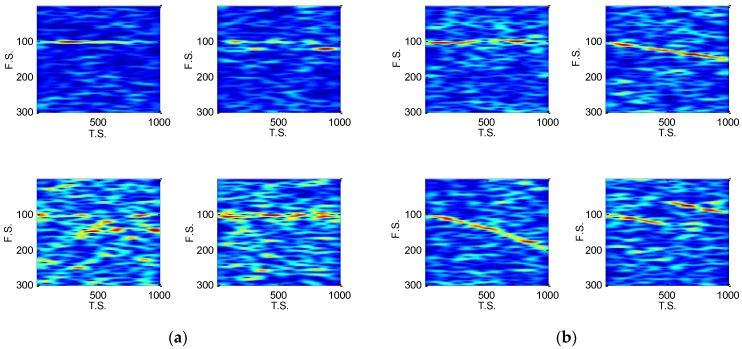
The TFD of eight types of signals at −5 dB: (**a**) STFT of NS, BFSK, QFSK and BPSK; (**b**) STFT of QPSK, LFM, NLFM and FRANK.

In [3], the radar emitter recognition based on STFT and CNN is discussed. It indicates that the recognition between NS and BPSK, BFSK and QFSK are not good at a low SNR. The effect of noise decreases the difference between the images, and the denoising cannot solve it well. Figure 3 shows the STFT of eight types of signals at −5 dB. From the images, the NS, BPSK and QPSK’s subtle features are covered by the noise and it was difficult for CNN model to extract the similar time-frequency image features at low SNRs, such as NS and QPSK, QFSK and BPSK in Figure 6. Because of the AGWN, other confused recognition might appear in the simulations, too, so it is important to reconstruct the distinctive features of each intra-pulse signal.

#### 4.1.2. Energy Cumulant of Short Time Fourier Transform

The TFD is a good method to study radar emitter intro-pulse signals. In [21,22,23], STFT, WVD and CWD were improved and applied to recognition work. In [3], we combined STFT with CNN to complete the recognition task and found that the noise could disorder the two dimensional distribution, which resulted in a low recognition rate of less than −3 dB. Then we tried to find a feature based on STFT to represent the signal even at a low SNR. In [37], Zhang extracted the edge of STFT images and got the projection after accumulation at the y-axis. However, it was considered at a SNR no less than zero. When the SNR continues decreasing, the edge extraction results will deteriorate. We analyzed the STFT with noise and discovered that, although the noise influences the signal’s TFD, it is restricted to a part of the pixel samples. After denoising, the signal’s TFD loses some energy in pixels, while most of the energy remains. The two dimensional STFT supplies more information than the time or frequency domain features, and it also scatters the noise and makes the denoising more effective. Each pixel can be regarded as one energy point and the signal pixels of the main interval are obviously larger than the noise. We used the time-frequency domain to remove most of noise and preserve the main TFD of signals. Therefore, we tried to find a transform based on the remained TFD of signals to establish a new feature. When each energy point cumulates together at one frequency point with all the time sequence, the needed feature is reconstructed in new form via preprocessing. The energy cumulant of STFT is defined by:

(8) $$ECSTFT_{x}(n)=\sum_{m=1}^{p}\left|STFT_{x}(m,n)\right|,n=1,\;2,\;\ldots ,\;q,$$

where the |⋅| is the absolute value operation, *m* is the time sequence, *n* is the frequency sequence, p is the length of time sequence in STFT, q is the length of frequency sequence in STFT. The computation code of ECSTFT can be found in the Section Appendix A. Besides, the lengths p and q can be changed by sampling the STFT results to decrease the calculation and simplify the learning process.

**Figure 4:**
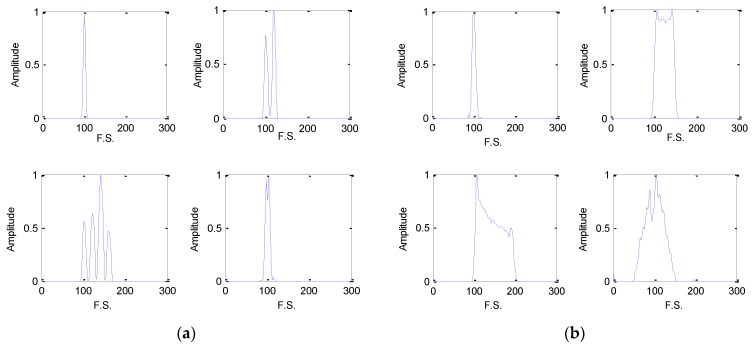
The EC-STFT of eight type radar emitter intra-pulse signals at 20 dB, and the 300×1000 image has been sampled into 300×100 size, p=100,q=300: (**a**) EC-STFT of NS, BFSK, QFSK and BPSK; (**b**) EC-STFT of QPSK, LFM, NLFM and FRANK.

**Figure 5:**
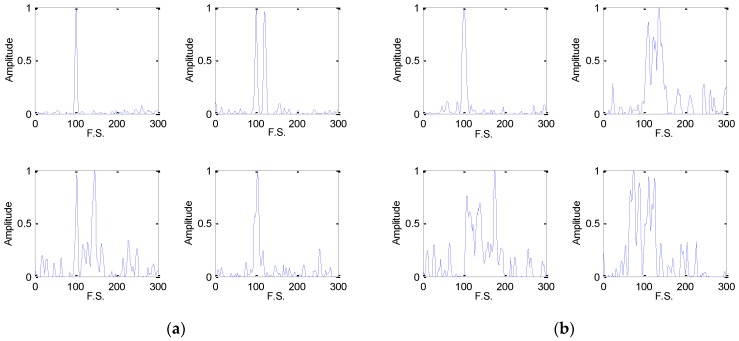
The EC-STFT of eight type radar emitter intra-pulse signals at −5 dB: (**a**) EC-STFT of NS, BFSK, QFSK and BPSK; (**b**) EC-STFT of QPSK, LFM, NLFM and FRANK.

Figure 4 and Figure 5 are the EC-STFT of the eight type radar emitter intra-pulse signals at 20 and −5 dB obtained via preprocessing. According to Figure 4, the NS, BPSK and QPSK contain their own differences: the peak of NS is the narrowest; the BPSK has a fork; and the QPSK is widest.

In Figure 5, the noise still exists, but the location of the peak or band still provides enough diversities and the RDBN can use these diversities to complete the recognition task at a low SNR. Besides, the display of EC-STFT in Figure 4 is similar to the frequency spectrum generated by FT of each intro-pulse signal. Theoretically, the STFT shows the variation of frequency with time and FT is a global transform. In FT, the amplitude represents the energy of one frequency point, while in STFT image, the value of each pixel represents the energy of one frequency point at one time point. When we added all the pixels of the time sequence of one frequency, we attained the amplitude of different frequency points that are similar to frequency spectrum. As a result, the EC-STFT has similar properties as FT.

### 4.2. Feature Preprocessing

The preprocessing is before the feature vector or matrix formed as input to the recognition network, and it aims to keep intra-pulse signals recognized correctly by the trained network. So some measures are taken as follows:

- The carrier frequency $f_{0}$ of the intra-pulse signal is the intermediate frequency. When it is at other frequency points, it will be moved to $f_{0}$.
- Suppose $A_{N\times N}$ is the TFD matrix of a noisy radar emitter intra-pulse signal via STFT in a large size. And a cutting and sampling on $A_{N\times N}$ are conducted with the result of $A_{p\times q}$. To process the noise, $A_{p\times q}$ is transformed into a vector, $\alpha =[\alpha_{1},\alpha_{2},\ldots ,\alpha_{p}]$, in which $\alpha_{i}$ is the raw vector of $A_{p\times q}$. For α, a zero-mean scaling is completed by:
  (9) $$\alpha^{\prime }=\frac{\alpha -mean(\alpha )}{var(\alpha )},$$
  where the mean(⋅) is mean of all the elements of α, and var(⋅) gets the variance of all elements of α. Then the denoising is operated by:
  (10) $$ϒ(\alpha^{\prime })\to \{\begin{array}{c} 0,(a_{i}<0)\\ a_{i},(a_{i}\geq 0) \end{array},1\leq i\leq p\times q,$$
  where $\alpha^{\prime }=(a_{1},\ldots a_{i},\ldots ,a_{p\times q})$ and $ϒ(\alpha^{\prime })$ removes the weak noisy points. Apparently, the Equation (10) just removes a part of noise. In order to get better denoising performance, the Equations (9) and (10) are repeated six times [3].

Then the processed vector $\alpha^{\prime }$ is transformed into a matrix ${\tilde{A}}_{p\times q}$. According to the Equation (8), ${\tilde{A}}_{p\times q}$ turns to be a new feature vector β via EC-STFT, and maximum normalization is conducted on β by:

(11) $$v=\frac{\beta }{max(\beta )},$$

where the max(⋅) refers to the maximum element of β which is input to RDBN as training set feature vector.

**Figure 6:**
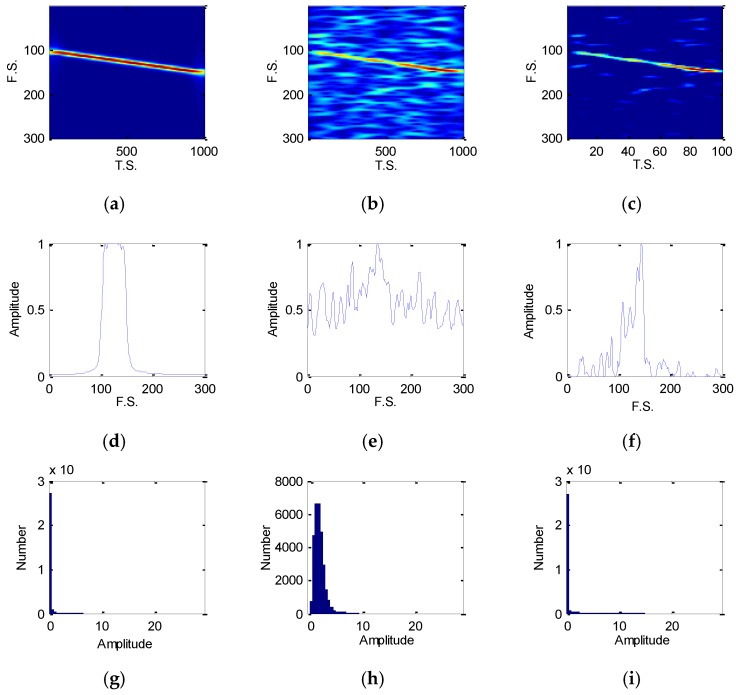
The results of STFT image, EC-STFT and the histogram of LFM signal: (**a**,**d**,**g**) for LFM signal without noise; (**b**,**e**,**h**) for LFM signal with noise (−5 dB); (**c**,**f**,**i**) for LFM signal after denoising.

Figure 6 shows the comparison of STFT image, EC-STFT and histogram of amplitude in EC-STFT before and after denoising on LFM signal. Comparing (c) to (a) and (b), most of the noise was removed and the features of time-frequency image were well preserved. From (d), (e) and (f), a part of the energy of the pixels in the STFT image was lost, but the EC-STFT still kept the significant features which could make the intro-pulse signal recognized by the model. Besides, the amplitude distribution in EC-STFT of (g), (h) and (i) had indicated that the denosing method is feasible.

### 4.3. Reinforced Deep Belief Networks

DBN is one for the first non-convolutional models successfully applying deep architecture training [27,28]. In [27], Hinton proposed a generative model using a fast, greedy algorithm for initialization and a contrastive vision for fine-tunes, which gives better digit classification than the best discriminative learning algorithms. In [29], Ling presented a new spectral modeling method using Restricted Boltzmann machine (RBM) and DBN for statistical parametric speech synthesis and improved the naturalness of the conventional HMM-based speech synthesis system. In [30], Yang proposed a competitive DBN to learn features with more discriminative information from labeled and unlabeled samples for underwater acoustic target recognition and got the higher classification accuracy. The DBN-based algorithms commonly concentrate on improvement of the exterior structure. We have studied initial training of the RBM in DBN model, and discovered the values in the weighted matrix related to the significance of the visible layer. Then we applied the idea of reinforce learning to revise the weighted matrix inside the DBN. The RDBN is combined with the new formed feature vector to achieve the recognition task.

#### 4.3.1. Restricted Boltzmann Machine and Deep Belief Network

**Figure 7:**
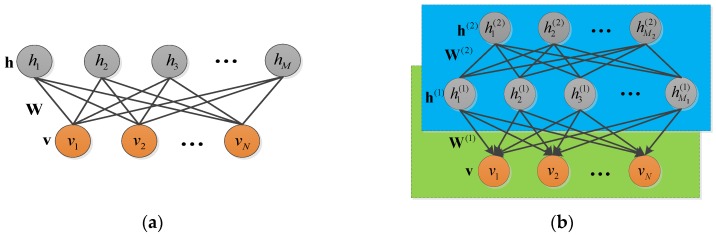
The model represented by RBM: (**a**) RBM; (**b**) DBN with two hidden layers.

RBM is an undirected probability graph model based on the energy with a visible layer and hidden layer [26]. Figure 7 gives the RBM’s architecture in (a). The visible layer is composed of *N* input variables, $v=(v_{1},v_{2},\ldots ,v_{N})$; and hidden layer is composed of M input variables, $h=(h_{1},h_{2},\ldots ,h_{M})$. In the architecture, each visible unit connects to the hidden units with the weighted parameter ***W***, and the same layer remains unconnected. Supposing $v_{i}\in \{0,1\}$, $h_{j}\in \{0,1\}$, the joint probability distribution of **v** and **h** is decided by:

(12) $$P(v,h)=\frac{1}{Z}exp(-E(v,h)),$$

where *Z* is the normalized constant:

(13) $$Z=\sum_{v}\sum_{h}exp(-E(v,h)),$$

and the energy function is defined by:

(14) $$E(v,h)=-\sum_{i=1}^{N}a_{i}v_{i}-\sum_{j=1}^{M}b_{i}h_{i}-\sum_{i=1}^{N}\sum_{j=1}^{M}w_{ij}v_{i}h_{j},$$

where $a_{i}$ and $b_{j}$ are the bias of **v** and **h**, $w_{ij}$ is the weight between $v_{i}$ and $h_{j}$, and **W** is the weight matrix between visible layer and hidden layer.

In this work, the visible layer input **v** is real value and hidden layer input is binary, so the RBM uses the Gaussian-Bernoulli form. And the energy function is defined as:

(15) $$E(v,h)=-\sum_{i=1}^{N}\frac{{\left(v_{i}-a_{i}\right)}^{2}}{2\sigma_{i}^{2}}-\sum_{j=1}^{M}b_{i}h_{i}-\sum_{i=1}^{N}\sum_{j=1}^{M}w_{ij}\frac{v_{i}}{\sigma_{i}}h_{j}.$$

According to [26,27,28,29], the conditional distribution P(h|v,θ) and P(v|h,θ) are:

(16) $$P(h_{j}=1|v,\theta )=s(b_{j}+\sum_{i}v_{i}w_{ij}),\;P(v_{i}=1|h,\theta )=N(a_{i}+\sigma_{i}\sum_{j}h_{j}w_{ij},\sigma_{i}{}^{2}),$$

where s(x)=1/(1+exp(−x)), and $Ν(\mu ,\sigma^{2})$ is the Gaussian distribution. The variance parameters $\sigma_{i}{}^{2}$ are commonly fixed to a predetermined value instead of learning from the training data [26,28], and we valued $\sigma_{i}{}^{2}=1$ for convenient computation. The RBM parameter θ={a,b,W} is trained by the contrastive divergence (CD) algorithm. By the training data, the θ is updated as:

(17) $$\Delta w_{ij}=[E_{D}(v_{i}h_{j})-E_{M}(v_{i}h_{j})]\cdot \alpha$$

In Equation (17), $E_{D}$ refers to the expected value of observation, $E_{M}$ is the expected value of defined distribution after model established, and α is the learning factor. Besides, the offset updating $\Delta a_{i}$ and $\Delta b_{j}$ are calculated in the same way.

DBN is composed of many hidden layers [25,27,29,30], and Figure 7b gives the two-hidden-layer DBN. In the DBN model, it refers to the mixture of directed and undirected connections. The top two layers are undirected connected, and the others are directed. The *L*-layer DNB has *L* weighted matrixes: $W^{\left(1\right)},W^{\left(2\right)},\ldots ,W^{\left(L\right)}$, L+1 offset vectors: $a^{\left(0\right)},a^{\left(1\right)},\ldots ,a^{\left(L\right)}$, and $a^{\left(0\right)}$ is the offset of visible layer. The probability distribution in DBN is defined by:

(18) $$P(h_{i}{}^{\left(l\right)}=1|h^{\left(l+1\right)})=s(a_{i}{}^{\left(l\right)}+W_{:,i}^{\left(l+1\right)}{}^{T}h^{\left(l+1\right)}),\;P(v_{i}=1|h^{\left(1\right)})=s(a_{i}{}^{\left(0\right)}+W_{:,i}^{\left(1\right)}{}^{T}h^{\left(1\right)}),$$

where l=1,2,…,L, s(⋅) is the sigmoid function. When v is real valued, the visible layer and the first hidden layer can be described by Equation (16). In DBN, the former layer is regarded as the visible layer of the upper hidden layer, and these two are composed of a RBM. When the first RBM training is completed, the network parameters are kept and the next RBM training task is executed until it gets to the top layer. In DBN training procedure, the unsupervised learning is used to train the former RBMs, and the supervised learning completes the top layer classifier and achieves optimization by the BP algorithm.

#### 4.3.2. Reinforced Deep Belief Network

**Figure 8:**
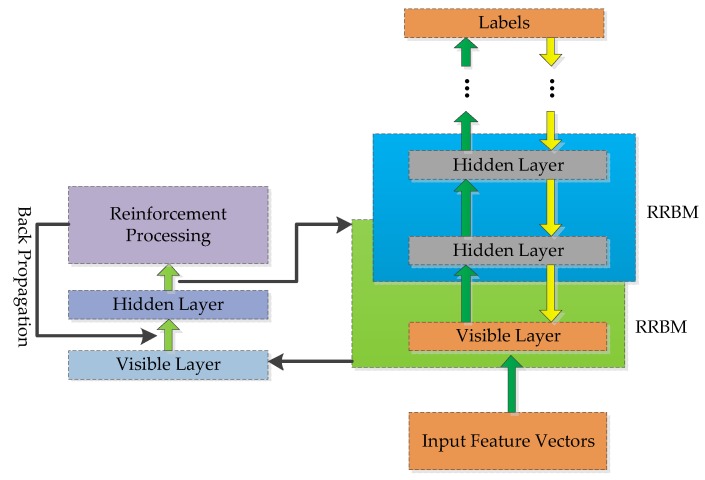
The structure of RDBN.

The structure of RDBN is presented in Figure 8. The RDBN training process is similar to the DBN, and they both combine the unsupervised learning and supervised learning in the training procedure. Firstly, the former training process uses unsupervised learning to get the initial RBM network parameters. Secondly, the reinforcement learning concept is integrated into the trained RBM to establish the reinforced RBM (RRBM). Thirdly, these RRBMs compose the RDBN in a stack, and the next supervised learning finishes the recognition network training with the labels connected to the top layer by BP algorithm.

**Figure 9:**
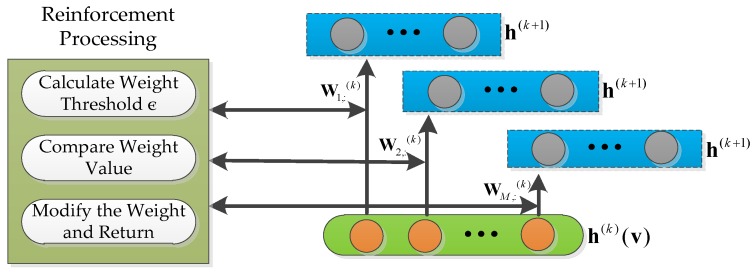
The framework of RRBM.

The weight matrix in RRBM is trained to complete the representation between adjacent layers. In the representation, the distribution of weight matrix $W^{\left(k\right)}$ reflects the characteristics of the input emitter signal data. After the unsupervised training, the connected weight matrixes are kept and the later supervised training will complete the final distribution of $W^{\left(k\right)}$ which can recognize the eight types of radar emitter signals. During our studies on $W^{\left(k\right)}$, we found that different $w_{ij}^{\left(k\right)}$ worked differently in the distribution. The larger the absolute value of $w_{ij}^{\left(k\right)}$ is, the more important the $h_{i}^{\left(k-1\right)}$ ($v_{i}$) of the visible layer is to the $h_{j}^{\left(k\right)}$ in the hidden layer, so the RRBN concentrates on processing the connected weight matrix after finishing unsupervised learning in each RBM as Figure 9 shows. The reinforcement processing is applied to $W^{\left(k\right)}$, and it includes three parts: firstly, calculate the threshold ε to the each row of $W^{\left(k\right)}$; secondly, compare the ε with every weight value $w_{ij}$; thirdly, modify the $w_{ij}$ according to the comparing result and return the value. In this procedure, the concrete modification algorithm is shown in Algorithm 1, and all the output ${\hat{W}}^{\left(k\right)}$ will replace the old connected weight matrix $W^{\left(k\right)}$ for the next supervised learning.

**Algorithm 1.** The reinforcement algorithm on $W^{\left(k\right)}$ in RRBN.**Input**: Weight matrix $W^{\left(k\right)}$;          Learning rate α;          Tuning factor ρ,γ.
**Process:**
1. $\varepsilon =[\varepsilon_{1},\varepsilon_{2},\ldots ,\varepsilon_{N}]$, $W^{\left(k\right)}=\{w_{ij}^{\left(k\right)}\}$, ${\hat{W}}^{\left(k\right)}=\{{\hat{w}}_{ij}^{\left(k\right)}\}$;2. $\forall i=1,2,\ldots ,N;j=1,2,\ldots ,M:\varepsilon_{i}=\frac{1}{M}\sum_{j}\left|w_{ij}^{\left(k\right)}\right|$
3.  **for**
i=1,2,…,N
**do**4.   **for**
j=1,2,…,M
**do**5.    **if**
$|w_{ij}^{\left(k\right)}|>\varepsilon_{i}$
**do**6.        ${\hat{w}}_{ij}^{\left(k\right)}=w_{ij}^{\left(k\right)}+sign(w_{ij}^{\left(k\right)})\cdot \alpha \cdot \rho$;7.    **else**8.        ${\hat{w}}_{ij}^{\left(k\right)}=w_{ij}^{\left(k\right)}-sign(w_{ij}^{\left(k\right)})\cdot \alpha \cdot \gamma$;9.    **end if**10.  **end for**11. **end for****Output**: Reinforced weight matrix ${\hat{W}}^{\left(k\right)}$.

## 5. Simulations and Discussions

The efficiency and robustness of the proposed method are analyzed and evaluated by simulations. In this part, three main tasks are conducted: firstly, the recognition network RDBN is validated and the network parameters are analyzed; secondly, the new generated feature EC-STFT fed to the recognition model is discussed and tested; thirdly, the overall performance of the proposed method is displayed and compared with other approaches.

The established recognition framework focused on the eight types of radar emitter intra-pulse signals: NS, BFSK, QFSK, BPSK, QPSK, LFM, NLFM and FRANK. As for the signal parameters, they are set at the same intermediate frequency: 10 MHz, the sampling frequency is 100 MHz, the pulse width (PW) τ is 10 μs; the frequency hopping of BFSK and QFSK is 2 MHz; the bandwidth (B) of LFM and NLFM is 5 MHz; the LFM slope is 0.5 MHz/μs; the NLFM adopts the polynomial ($f(t)=B\cdot t/\tau +B\cdot t^{2}/\tau^{2}$); the code of BPSK, QPSK and FRANK are ‘0,1,0,1,1,1,0,0,1,0’, ‘3,0,1,2,2,1,3,2,1,0’ and FRANK (8). The training set is based on the EC-STFT of the signals in high SNR (30 to 40 dB), and these data support the RDBN training. The testing set includes a range from −10 to 5 dB of data to validate the work. The signals are mixed with additive white Gaussian noise (AWGN). In the fundamental simulation, the length of time domain signal is 1000, and the length of feature vector via EC-STFT is 100; the training set size is 4000, and each type contains 500 samples and labels; the testing set size contains 200 samples at one SNR value; and the epoch number is 1, the batch size is 5, learning factor (α) is 0.1. After the RDNB finishes the training process, the testing set is input for recognition, and the Monte Carlo experiments are conducted 1000 times. We compare the output labels with the testing labels and calculate the correct recognition rate. The single recognition rate (SRR) and average recognition rate (ARR) at a SNR value are defined by:

(19) $$SRR=\frac{1}{K}\sum_{i}^{K}\frac{N_{s_{c}}^{i}}{N_{s_{{}_{t}}}^{}},\;\;ARR=\frac{1}{K}\sum_{i}^{K}\frac{N_{a_{c}}^{i}}{N_{a_{{}_{t}}}^{}}.$$

where K is the times of Monte Carlo, $N_{s_{c}}$ is the single type correct recognition number, $N_{s_{t}}$ is the single type total number, $N_{a_{c}}$ is the total correct recognition number of all types, $N_{a_{t}}$ is the total number of all types. And the influence of SNR environment, RDBN parameters and EC-STFT characteristics on recognition rate, the recognition results compared with other methods are presented as follows.

### 5.1. The Network Validation

**Figure 10:**
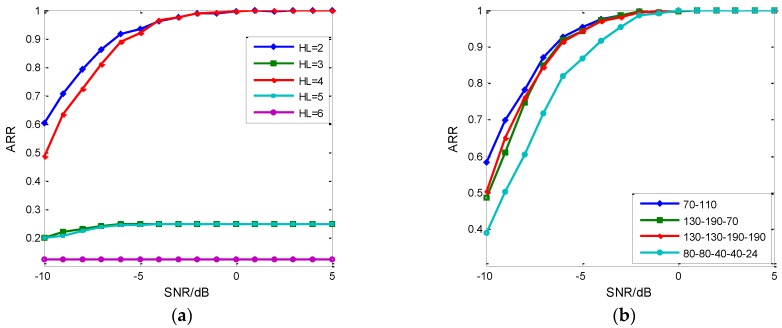
ARR of different hidden layers. (**a**) Each hidden layer has 200 nodes in RDBN; (**b**) each hidden layer has the tuned nodes in RDBN.

The RDBN can get the subtle features of the intra-pulse signal based on the EC-STFT. Each layer in RDBN achieves the data representation. The selection of layers has important effect on the final recognition results. In the simulation, we set input vector length 100, hidden layer nodes 200 for each. Figure 10a shows the ARR of different hidden layers (HL) (from 2 to 6) with SNR changing. From Figure 10a, the two hidden layers get the best performance, and the next best is four hidden layers. It doesn’t present good recognition results in the other three architectures. Obviously, when the number of nodes in each hidden layer is set the same, more layers do not always achieve better performance. More layers can bring larger accumulating losses via the representation between layers. Further, the losses cause confused feature distributions and decreased RDBN recognition. Besides, the decreasing SNR also increases the difficulty of the recognition work. Of course, this does not mean the multi-layer concept makes no sense. It depends on the input vector’s length and the tuning of each layer’s nodes. When the input vector’s length is confirmed, every hidden layer’s nodes should be adjusted to the input. The three, five and six hidden layers with 200 nodes in each are apparently unsuitable for the input vectors in RDBN. Figure 10b gives the considerate optimal solution of recognition results after adjusting the node parameters. According to the results, the recognition results of three and five hidden layers improve a lot more than before, while the two hidden layers case still remains the best.

In the work, the multiple hidden layers also bring about the increasing training time problem. An AMD Athlon (TM) II X3 445 CPU with 4 + 4 GB processor (3.1 GHz) was used in the simulations. Table 1 records the training times of the different architecture in Figure 10a,b. Apparently, when the node number of each layer is set the same, the increasing layers produce increasing node-to-node calculations.

However, the tuned nodes in each layer have changed this phenomenon. According to Table 1, the training time maintains a linear relation with the number of layers. When the nodes decrease in one layer, the training time also decreases sharply.

**Figure 11:**
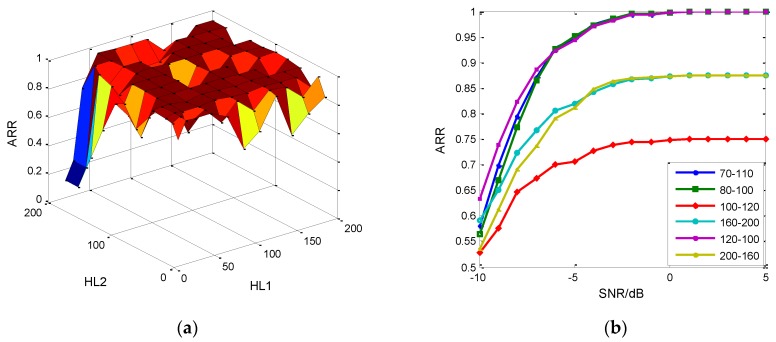
The parameter tuning of 2 hidden layer RDBN. (**a**) Effects on the recognition rate at −5 dB of nodes in hidden layers; (**b**) Examples of parameter tuning selected from the simulations.

From Figure 10, the RDBN with two hidden layers has a similar performance compared with other architectures. Besides, the 2-hidden-layer architecture is the simplest and it costs the least training time, so we concentrated on analyzing the effects of node parameters on the two-hidden-layer RDBN’s recognition performance. In common, the parameter tuning is finished by experience operation, while we conducted the traversal search on the two-hidden-layer RDBN. The result is shown in Figure 11a. 

In the simulations, the nodes in the two hidden layers are constrained from 20 to 200 with a step size of 20. We selected the recognition results at −5 dB as the object of comparison. The best architecture is 80–100 with a 95.06% recognition rate in the simulations. Also, other combinations can achieve similar performance. Figure 11b enumerates six different combinations, and half of them attain good results in which 120–100 becomes the optimal one below −6 dB and 70–110 was selected manually that did not appear in the traversal search. The search takes about 4.5 h, while it cannot search for all the conditions. As for three to six hidden layers, it needs to consume much more resources. This parameter tuning originates from the generated networks, and the 70–110 is the fine tuning based on the 80–100. Hence, there could be other optimal combinations for selection, which do not present in the traversal search. In the following simulations, we select 70–110 combinations for RDBN.

**Figure 12:**
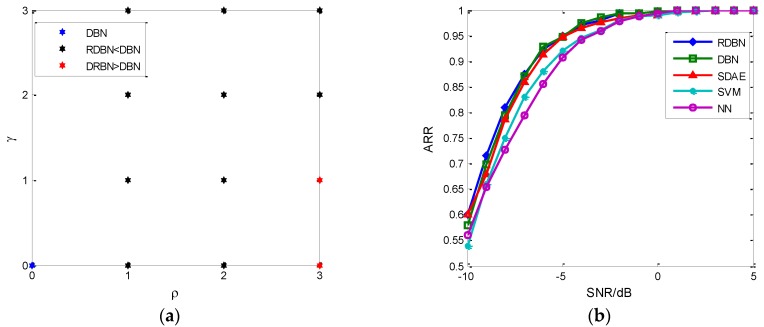
The comparison between DBN and RDBN. (**a**) The effect of tuning factor on SRR at −5 dB; (**b**) Recognition result of different recognition networks.

The reinforcement algorithm on $W^{\left(k\right)}$ in RDBN also improves the recognition performance. According to Figure 10, the algorithm’s performance is mainly decided by the tuning factors ρ and γ. Although both $W^{\left(k\right)}$ and ${\hat{W}}^{\left(k\right)}$ will be adjusted in the later supervised training, the distribution of weight can influence the final recognition result. Figure 12a shows the recognition result at −5 dB with different tuning factor combinations. The result of ρ=3, γ=1 and ρ=3, γ=0 get to the desired result. Furthermore, the tuning factors are varied with different hidden layers. For 120–100 combinations, the optimal tuning factors are ρ=1, γ=1. In Figure 12b, comparison between DBN and RDBN about their recognition performance based on 70–110 combinations and ρ=3, γ=1 is presented and other recognition networks, stacked denoising autoencoder (SDAE), support vector machine (SVM), neural network (NN), are also fed the new feature, EC-STFT. The result indicates that the proposed RDBN has an advantage over other networks in recognition. 

### 5.2. The Fearture Set Discussion

**Figure 13:**
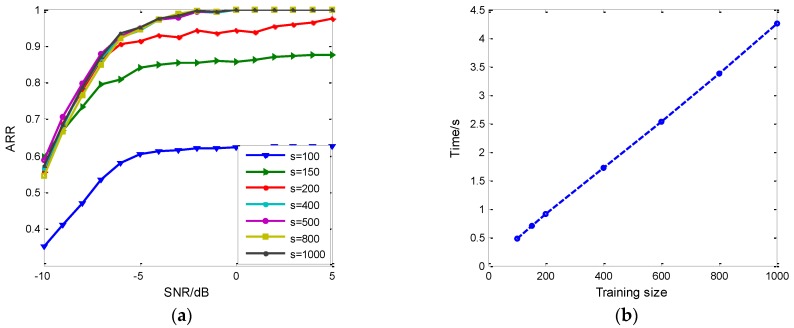
The effect of training size on RDBN performance. (**a**) The increase of training samples improves ARR within limits; (**b**) The training consuming time and sample size are linearly related.

In addition to the network architecture and parameters, the feature set influences the recognition performance in the proposed method, too. As one of the deep generation models in deep learning, RDBN needs the support of enough training samples. The large training set keeps the generated network learning more sufficient and makes the recognition more discriminative. The training set is generated by the high SNR signals, and the testing set is under low SNR, so the over-fitting phenomenon can be ignored and the robustness of the method can be evaluated. Figure 13a shows the recognition results of different training size (*s*) as the SNR varies, where *s* represents the sample number of each type signal. When *s* increases, the ARR improves; when *s* comes to a threshold, the ARR will stay nearly unchangeable. From Figure 13a, use of 400 samples of each type of emitter intra-pulse signal for training can achieve as good performance as the 500 to 1000 sample training set. However, when it refers to the training consuming time, the time is linearly related to the size of the training set, as proved by Figure 13b. In the fundamental simulation, the training size selected was 500 to balance the ARR and training time.

**Figure 14:**
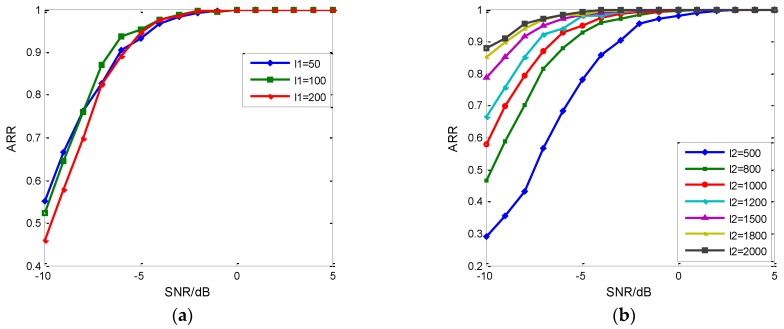
The influence of sample length on ARR. (**a**) The influence of input feature vector’s length on the ARR; (**b**) The influence of time domain signal’s length on ARR.

The input feature vector is also important to the establishment of the RDBN. The more useful information and more unique characteristic it provides, the better performance the RDBN will have. Figure 14 gives the recognition based on training samples with different properties. In (a), the length of the time domain signal is 1000, and the new feature vector’ length is 500 after EC-STFT. We made an equal interval sampling on the new feature vector to get the three length ones: 50, 100 and 200. Although the longest one does not get the best result, the results of 100 and 200 are than 50’s. In (b), the length of the input vector is set 100 after sampling, and the increase of the time domain signal’s length improves ARR. Besides, the improvement is decreasing gradually. With the decreasing SNR, the sampled values from the EC-STFT contain noise pollution. According to Equation (8) and Figure 4, no more key values are supplied by increasing the sampling values when the time domain signal’s length is constant; on the contrary, it provides an extra key value by increasing the time domain signal’s length when the input vector’s length is constant, so when establishing the input vector, the sampling in the time domain and transformed domain both need to be considered.

From Figure 4 and Figure 5, the EC-STFT is indeed similar to the FT of a time domain signal, but they are quite different with the noise processing. The EC-STFT eliminates its noise in the time frequency domain, while the FT does so in the time/frequency domain. The noise distribution in the two- dimension transformed domain is more dispersive than the FT’s, so the noise reduction in the frequency spectrum is more difficult than it in the time frequency domain and it only relies on the time domain processing.

**Figure 15:**
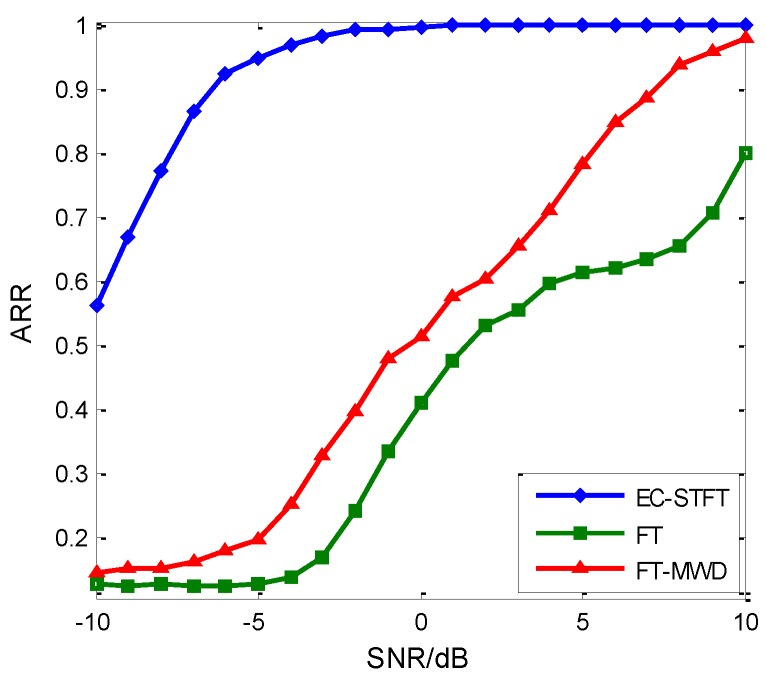
The comparison between EC-STFT and FT as feature selection

We have proposed a noise reduction method based on wavelet transform aiming at radar emitter intra-pulse signals in [38], but only to solve part of the problem. We applied the EC-STFT and FT features to the RDBN for recognition experiments. Figure 15 shows the recognition results based on EC-STFT, FT and FT-WMD, and the EC-STFT-based one apparently gets the best recognition result. Comparing Figure 12b with Figure 15, the EC-STFT-based fed to other networks are obviously better than FT-based fed to RDBN. As a result, the EC-STFT contributes more than RDBN to our proposed recognition method.

**Figure 16:**
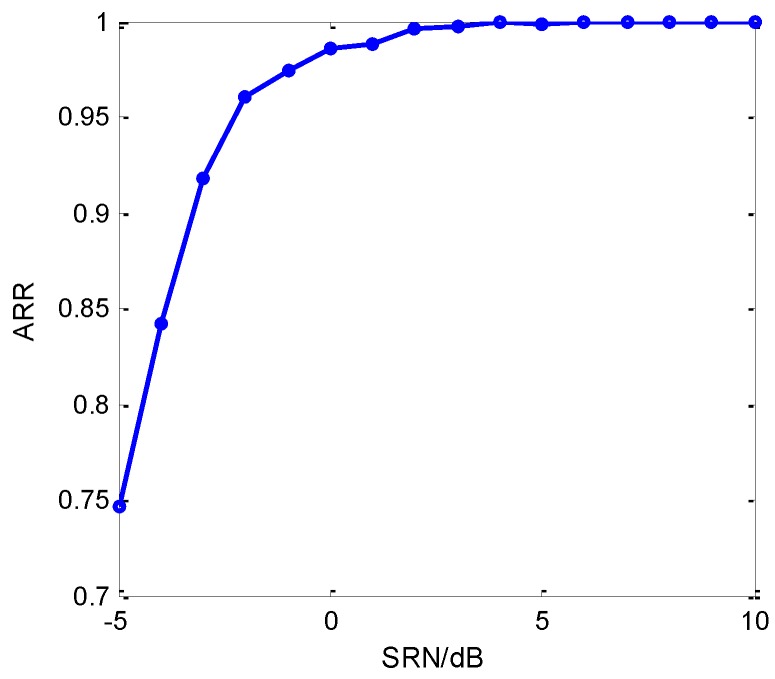
ARR of the proposed method with different slopes on LFM.

The former simulations mainly concentrate on the inter-class recognitions, and next the intra-class recognition will be examined. Then, the slopes of LFM are set 0.5 MHz/μs (LFM1), 1.0 MHz/μs (LFM2) and 1.5 MHz/μs (LFM3), and other types of intra-pulse signals remain the same according to the parameter settings used before. For these three LFMs, 500 training samples of each are added in the training set and 200 samples of each at different SNR are added for testing. Besides, the tuning of node number of two hidden layers is 240–320 and ρ=3, γ=1. Figure 16 shows the recognition results of the proposed method on the new testing set. 

From Figure 16, the recognition performance based on EC-STFT and RDBN decreased more than the former. In fact, the similar bandwidth between LFM2 and NLFM caused this phenomenon. When LFM2 and NLFM have the same intermediate frequency and similar bandwidth, recognition on them is easily influenced by the environment noise. Table 2 presents the confusion matrix of the recognition result with different LFMs at −2 dB. Apparently, SRR of LFM2 and NLFM are lower than others, and their recognized errors are mainly focused on themselves and the problem is getting worse with decreasing SNR, although, according to Figure 16 and Table 2, the proposed method still has a good recognition ability.

### 5.3. The Method Performance Comparison

**Figure 17:**
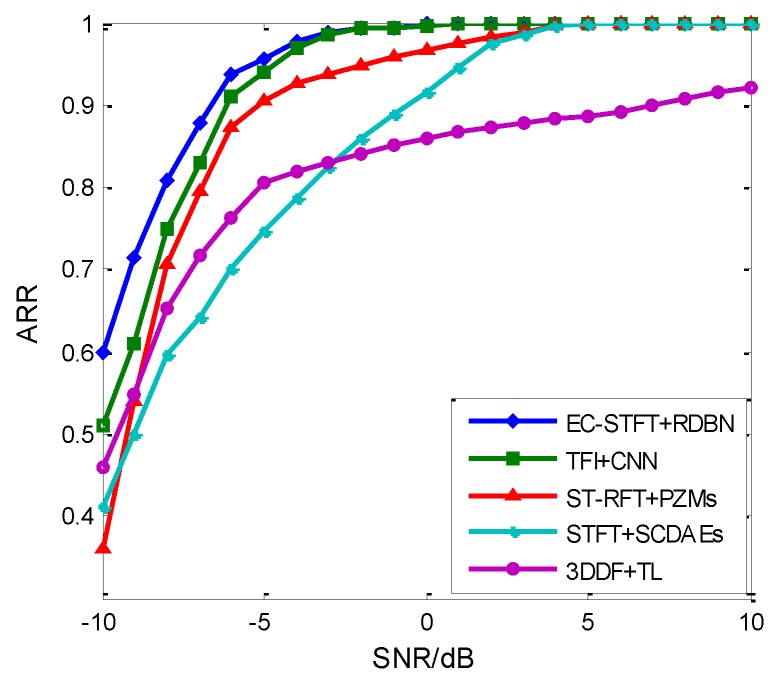
The comparison of different radar emitter intra-pulse signal recognition methods.

The proposed method based on EC-STFT and RDBN achieves a higher recognition rate than other radar emitter intra-pulse signal recognizing methods. In the simulations, we selected four recognition methods with similar study objects and constraints: STFT + SCDAEs [2], 3DDF + TL [21], TFI + CNN [22] and ST-RFT + PZMs [23]. These four methods all focus on radar emitter intra-pulse signal recognition with five to seven types. Figure 17 shows the recognition results with varying SNR of the approach proposed in this work and the other four methods. According to the results, all except 3DDF + TL present a nearly 100% recognition rate over 5 dB, and EC-STFT + RDBN and TFI + CNN even achieve it at −2 dB. Besides, the recognition performance of EC-STFT + RDBN presents better than TFI + CNN below −5 dB, and our work gained a 95% recognition rate at −5 dB using the fundamental experiment conditions. Figure 14b, it shows that the recognition rate can increase continuously as the time samples increase. In all, the proposed method in this work demonstrates a better performance in recognition tasks.

## 6. Conclusions

In this paper, a method based on EC-STFT and RDBN was proposed for radar emitter intra-pulse signal recognition. The EC-STFT reserves most of the significant feature information of the intra-pulse signals even at a low SNR. During the training time, the RDBN dominates the feature extraction tending to benefit recognition with the reinforcement algorithm. Our experimental simulations show both the EC-STFT and RDBN contribute to the proposed recognition method which has better recognition performance than other approaches, while the EC-STFT contributes more.

As we displayed in Section 5, most of the simulation results were obtained under the fundamental condition. We conducted these simulations with finite sources, for example, the sampling length of time-domain signals, the pixels reserved in TFD and the structures of the recognition networks are all set for feasibility verification. Some simulation results indicated that when these conditions were unlimited, the recognition performance could be at a higher level.

This paper only explores the combination of one-feature and deep learning model for radar emitter recognition. There are still many issues to be discussed further. First, it is worth applying multiple features in the deep learning model for recognition work as we present in the Introduction section. Second, the format of the datasets fed to the different deep learning models needs to be considered. In our work, the input feature should be a vector. It is a good idea to find a pre-training algorithm to generate the format we want.

## Figures and Tables

**Table 1:** The training time of the different hidden layers in Figure 10.

Layer	2	3	4	5	6
Time/s (a)	4.585	7.237	10.175	12.512	14.352
Time/s (b)	2.365	5.174	8.396	3.689	-

**Table 2:** Confusion matrix of SRR on LFMs with different slopes at −2 dB.

SRR (%)	NS	BFSK	QFSK	BPSK	QPSK	LFM1	LFM2	LFM3	NLFM	FRANK	Total
NS	**100**	0	0	0	0	0	0	0	0	0	100
BFSK	0	**100**	0	0	0	0	0	0	0	0	100
QFSK	0	0	**100**	0	0	0	0	0	0	0	100
BPSK	0	0	0	**99**	1	0	0	0	0	0	100
QPSK	0	0	0	5	**95**	0	0	0	0	0	100
LFM1	0	0	0	0	0	**100**	0	0	0	0	100
LFM2	0	0	0.5	0	0	0	**79.5**	1	**19**	0	100
LFM3	0	0	0	0	0	0	0	**100**	0	0	100
NLFM	0	0	1	0	0	0.5	**9**	0	**89.5**	0	100
FRANK	0	0	0	0	0	0	0	0	0	**100**	100

## Nomenclature

AOA	Angle of arrival
ARR	Average recognition rate
AWGN	Addictive white Gaussian noise
CD	Contrastive divergence
CFM	Continuous frequency modulation
CNN	Convolutional neural network
CWD	Choi-Williams distribution
DFM	Discrete frequency modulation
EC-STFT	Energy cumulant of short time Fourier transform
FT	Fourier transform
IF	Intermediate frequency
NN	Neural network
PA	Pulse amplitude
RBM	Restricted Boltzmann machine
RDBN	Reinforced deep belief network
RF	Radio frequency
RRBM	Reinforced restricted Boltzmann machine
SDAE	Stacked denoising autoencoder
SEI	Specific emitter identification
SRR	Single recognition rate
STFT	Short time Fourier transform
SVM	Support vector machine
TFD	Time-frequency distribution
TOA	Time of arrival
WVD	Wigner-Ville distribution

## Appendix A

**Algorithm A1.** Matlab Code of Computation on EC-STFT.
function [ecq,n] = ecstft(x)
% ECSTFT: Energy cumulation of short time Fourier transform
% x: signal.
% n: frequency sequence.
% ecq: the sequence of energy cumulation of short time Fourier transform
[tfr,t,f] = tfrstft(x); % The computation of STFT on x is based on the method proposed by Auger F., 1995
tfr_abs = abs(tfr);
q = length(t);
m = 1:q;
p = length(f)/2;
n = 1:p;
ecq = zeros(1,p);
for i = 1:p
ecq(i) = sum(tfr_abs(i,:));
end;
end;
